# Dysregulated Anxiety and Dysregulating Defenses: Toward an Emotion Regulation Informed Dynamic Psychotherapy

**DOI:** 10.3389/fpsyg.2018.02054

**Published:** 2018-11-05

**Authors:** Jon Julius Frederickson, Irene Messina, Alessandro Grecucci

**Affiliations:** ^1^Washington School of Psychiatry, Washington, DC, United States; ^2^Department of Philosophy, Sociology, Education and Applied Psychology, University of Padua, Padova, Italy; ^3^Department of Psychology and Cognitive Sciences, University of Trento, Trento, Italy

**Keywords:** emotion regulation, anxiety, emotion, defense mechanisms, dynamic psychotherapy, psychoanalysis

## Abstract

One of the main objectives of psychotherapy is to address emotion dysregulation that causes pathological symptoms and distress in patients. Following psychodynamic theory, we propose that in humans, the combination of emotions plus conditioned anxiety due to traumatic attachment can lead to dysregulated affects. Likewise, defenses can generate and maintain dysregulated affects (altogether Dysregulated Affective States, DAS). We propose the Experiential-Dynamic Emotion Regulation methodology, a framework to understand emotion dysregulation by integrating scientific evidence coming from the fields of affective neuroscience and Experiential-Dynamic Psychotherapy aimed at resolving DAS. This method and the techniques proposed can be integrated within other approaches. Similarities and differences with the Cognitive model of emotion regulation and cognitive-behavioral approaches are discussed within the paper.

## Introduction: Regulating Emotions From Neuroscience to Psychotherapy

According to Gross (1998), emotion regulation refers to “*processes by which individuals influence which emotions they have, when they have them, and how they experience and express these emotions*.” Theories of emotional regulation have their roots in the study of psychological defenses (Freud, 1936, 1959a,b; Paulhus et al., 1997), psychological stress and coping (Lazarus and Folkman, 1984), and theory of emotions (Frijda, 1986; Damasio, 1999; Ekman, 2003). Today, the field of emotion regulation integrates experimental research, clinical psychology, and neuroscience to study how emotions are generated and regulated to facilitate adaptation to the environment.

In traditional emotion regulation studies, participants are asked to apply a defined strategy when observing emotion-eliciting stimuli (regulation condition) or alternatively to observe the same emotional stimuli without applying any strategy (control condition). Results clearly show a marked reduction in the perceived intensity of emotional experience and dampened neural activation (for a review see Ochsner and Gross, 2005). Emotion regulation can affect the subjective emotional experience and the associated psychophysiological processes, such as heart rate, skin conductance,and neural activity (Jackson et al., 2000; Eippert et al., 2007). According to neuroimaging studies of emotion regulation (Ochsner and Gross, 2005; Buhle et al., 2013; Gross, 2014; Messina et al., 2015), the prefrontal cortex is thought to have an inhibitory effect on areas associated with emotional reactivity, such as the amygdala, resulting in decreased emotional experience in participants. Moreover, also temporal areas appear to be involved in emotion perception regulation, especially in the case of complex emotions in social situations in normal and abnormal populations (Grecucci et al., 2013a,b,c, 2016b; Grecucci and Sanfey, 2014; Pappaianni et al., 2018). These investigations into the regulation of social and complex emotions and the resulting emotion regulation theories provide a new understanding of mental disorders and their treatment.

After a decade of experimental studies, researchers have succeeded in gathering evidence to generate consensus about the key role of emotion regulation for healthy psychological functioning. The capacity to adaptively regulate negative emotion is considered a protective factor against the development and maintenance of psychopathology (Aldao and Nolen-Hoeksema, 2010; Aldao et al., 2010). On the other hand, difficulties in ER have been identified as putative risk and maintaining factors for several disorders (Gratz et al., 2013). Poor emotion regulation has been linked to psychiatric disorders (Barlow, 2002; Werner and Gross, 2010; Grecucci, 2012; Ehring, 2013; Mennin and Fresco, 2014; Messina et al., 2016a), and more than 75% of psychiatric disorders are characterized by deficits in emotion regulation (Kring and Werner, 2004). For example, anxiety, depression, and personality disorders are associated with specific dysregulated emotions (Thayer and Lane, 2000; Mennin and Fresco, 2009; Schulze et al., 2011). Moreover, emotion regulation strategies may differently influence psychological health. The most investigated strategy is reappraisal, an adaptive form of regulation that consists of the generation of new interpretations of stressful situations to decrease the emotional response they would otherwise elicit (Gross, 1998). Among the less adaptive strategies, suppression is one of the most investigated. It consists in controlling emotional expression, and it functions as a conscious defense mechanism (see Dysregulated Affective States Due to Defensive Affects). Compared to reappraisal, suppression has been negatively associated to the expression of positive emotion, effective interpersonal functioning, and well-being (Gross, 2002; Gross and John, 2003) and it negatively correlates with mental health indicators (Aldao and Nolen-Hoeksema, 2010; Hu et al., 2014). Taken together, these findings support the validity of a core construct of emotional dysregulation applicable to affective disorders and their therapy (Messina et al., 2013, 2016b). In line with this construct, techniques based on emotion regulation principles have been incorporated into cognitive-behavioral approaches. For example, Linehan (1993a,b) teaches a set of behavioral skills to help borderline patients cope with their dysregulated emotions. The implementation of cognitive and behavioral techniques – such as reappraisal and emotional avoidance – has been proposed as part of a unified protocol for proposed by Barlow et al. (2011). Moreover, specific emotion regulation trainings have been developed to treat emotional difficulties (Berking et al., 2008; Mennin and Fresco, 2009).

According to the experimental literature and the cognitive-behavioral tradition, emotions and anxiety are regulated by manipulating the thinking style (reappraisal strategy), or attention (distraction strategy) (Ochsner and Gross, 2005). The cognitive tradition has emphasized the conscious causes of emotion dysregulation and their conscious regulation and it does not clearly differentiate between what should be encouraged to express or to regulate. We believe that some psychodynamic principles can be used as a guide for the therapist to select what should be expressed or regulated. In the psychodynamic view, emotions are the fundamental way we make sense of the world. They tell us what we want and what we do not, what gives us pleasure and what gives us pain. They mobilize us to act adaptively on our own behalf, to pursue our goals in life (Tomkins, 1962). Like a GPS system, feelings tell us where we are, where we want to go, and how to get there (Frederickson, 2013). In everyday life, a stimulus triggers emotion (the “original emotion”), which is adaptive and proportionate to the stimulus, motivating the person to engage in adaptive behavior (Grecucci and Job, 2015). The function of conscious but also unconscious emotions as the primary motivators of behavior is widely recognized in psychoanalysis (Freud, 1959b; Kernberg, 1984, 1996), as well as in affective neuroscience (Panksepp, 1998; Damasio, 1999).

In psychodynamic therapy, patients are encouraged to experience emotions (sometimes even up-regulate them) and the associated impulse physically in the body, rather than downregulating them through cognitive or attentional strategies (Davanloo, 1990; Coughlin della Selva, 1996; Davanloo, 2000; Frederickson, 2013).

What should be regulated according to psychodynamic therapists by contrast, is the case of excessive anxiety or affects created by dysregulating defenses, so that the patient can be helped to express the underlying emotion (Hartmann, 1964).

In the present paper, starting from the above considerations, we propose that emotions, as evolutionary products, are not inherently dysregulated (as assumed by cognitive models of emotion regulation), and dysregulation can be better understood as the result of: (1) emotions plus dysregulated anxiety, or (2) defensive affects resulting from dysregulating defenses. We have defined these states as Dysregulated Affective States (DAS; Grecucci et al., 2015, 2016a). Second, we provide a framework and methodology for an Experiential-Dynamic Emotion Regulation Methodology (EDER) that integrates Experiential-Dynamic Therapy techniques and emotion regulation science principles for the clinical treatment of DAS.

## Dysregulated Affective States

### Dysregulated Affective States Due to Anxiety

Sometimes emotional responses are no longer proportional to the stimulus. Why? In some cases, the patient is overwhelmed by anxiety associated with a given emotion, and he/she may not be aware of the emotion covered by anxiety. According to the psychodynamic view, in such situations what becomes dysregulated and should be regulated is not the emotion itself, but *anxiety*. We call an emotion plus overwhelming anxiety a Dysregulated Affective State (DAS, Grecucci et al., 2015). Signal anxiety (Freud, 1959a) signals the emergence of relational and internal danger situations that would repeat earlier traumatic experiences. Psychodynamic theories describe how DASs originate in early relationships with caregivers, upon whom infants depend for their survival. Any emotion that triggers anxiety in the caregiver will be experienced as a danger to a relationship necessary for survival, causing a conflict between affect expression toward the caregiver and the need to be cared for by the caregiver (Sullivan, 1953; Bowlby, 1980). Through multiple experiences in early relationships, the infant, and later the child, learns unconsciously which emotions pose a danger to the relationship (Bowlby, 1980). Emotions trigger unconscious anxiety, based on memories of earlier conflictual relationships. Thus, anxiety becomes a conditioned response indicating that a rising feeling could endanger a relationship in the present, even though this is based on unconscious learning in the past (Bowlby, 1980). This psychoanalytic view of anxiety converges with neuroscience and neurobiology (Schore, 2003). According to affective neuroscience, when unconscious emotion rises, neuroperception of threat occurs triggering signal anxiety based on the inborn neurological fear circuit (Panksepp, 1998). Following the non-conscious perception of threat in the brain, a message is sent to the amygdala (LeDoux, 1998; Panksepp, 1998; Damasio, 1999). The amygdala activates the somatic and autonomic nervous systems, mobilizing the body to deal with a threat (Robertson et al., 2004; Porges, 2011).

When anxiety is discharged into the autonomic nervous system, it can be channeled into either the sympathetic or the parasympathetic nervous systems (Robertson et al., 2004) (see Table 1). As shown in Table 1, the sympathetic nervous system creates the symptoms of increased heart rate, blood pressure, and respiration; sweating, cold hands and feet, dry mouth, fainting from hyperventilation, and blushing. The parasympathetic nervous system creates the symptoms of decreased heart rate, blood pressure, and respiration; nausea, vomiting, diarrhea, migraines, dizziness, blurry vision, ringing in the ears, limpness, bodily anesthesia, and difficulty thinking. When patients experience emotions accompanied by the anxiety symptoms of the parasympathetic nervous system, they become dysregulated. They are not dysregulated by the emotion, but by the severe anxiety symptoms, including problems thinking and loss of reality testing. The problems of thinking and reality testing might seem unrelated to anxiety. However, activation of the parasympathetic nervous system releases neurohormones which inhibit the functioning of the hippocampus, responsible for storing short term memory (Sapolsky et al., 1990), and the prefrontal cortex, essential for higher order thinking (Wehrenberg and Prinz, 2007).

**Table 1:** Emotion dysregulation as a function of the pathways of anxiety discharge.

	Path of anxiety discharge	Symptoms/Signs	Level of emotional dysregulation	Therapeutic action
Emotion without anxiety	NA	NA	No dysregulation	Express feeling

Emotion plus mild anxiety	Somatic nervous system	-Hand clenching-Tension in the intercostal muscles of the chest, the patient sighs	No dysregulation	Watch anxiety while encouraging the patient to express feeling
		-Tension in arms, shoulders, neck, legs and feet		
		-Jaw clenching, biting…		

Emotion plus dysregulating anxiety	Parasympathetic nervous system	-Bladder urgency and frequency-Gastrointestinal spasm	Moderate to severe dysregulation	Regulate anxiety and then explore feeling
		-Irritable bowel syndrome, nausea, vomiting		
		Vascular—migraine, hypertension		
		Bronchi—asthma		
		“Jelly legs”		
		-Drifting		
		-Dissociation		
		-Confusion		

Emotion plus dysregulating anxiety	Cognitive- Perceptual system	-Hallucinations-Dissociation	Severe dysregulation	Regulate anxiety and then explore feeling
		-Blocking of thought		
		-Tunnel vision		
		-Tinnitus		


Notably, the pathway of anxiety discharge informs the therapist on how to intervene (see Table 1). In sum, when anxiety is mild, emotions can be encouraged. When anxiety is too high, the therapist needs to regulate the resulting DAS before getting into the underlying emotion. With the therapist’s help, the patient can become aware of these physical activations as emotions (Damasio, 1999). Once the patient is conscious of her emotions, those conscious emotions can mobilize conscious adaptive action (Damasio, 1999), and anxiety becomes regulated.

### Dysregulated Affective States Due to Defensive Affects

Much as we would like anxiety to be regulated easily, in many cases anxiety does not come down. Why? The patient may use defense mechanisms which create or perpetuate dysregulated affects. In earlier relational experiences the patient learned to avoid or cover certain emotions (e.g., anger toward the mother) through defenses, thus hiding feelings that might endanger a relationship (Sullivan, 1953). In a previous Section “Introduction: Regulating Emotions From Neuroscience to Psychotherapy,” we described an integrative theory of emotions as the primary motivators of behavior. Then we showed how anxiety is a biophysiological activation of the body paired through conditioning with emotions, and how dysregulated anxiety can create dysregulated affects (see Dysregulated Affective States). Now we will show how dysregulating defenses create dysregulated defensive affects.

In psychodynamic theory, defenses are understood as unconscious psychological mechanisms that reduce anxiety arising from unacceptable or potentially harmful thoughts, emotions or impulses (Freud, 1959a,b). Defenses are forms of adaptation to the environment that act by protecting individual self-esteem or self-integrity (Hartmann, 1964; Cramer, 1998), or important relationships (Sullivan, 1953; Bowlby, 1980). Although defenses are useful adaptations to damaging environments, once they become conditioned reactions to emotions or anxiety, they become generalized to other environments where they are unhelpful and even harmful. Thus, defenses which are useful in the here and now are adaptive. But, when they become conditioned reactions which are generalized, they create dysregulated defensive affects, or secondary affects, which cover or replace the original emotion. These defensive affects create a second type of DAS (due to the intervention of defenses).

When a stimulus occurs, we react with emotions. For example, if a boss does not give a promotion, this may trigger anger, and anger may trigger adaptive action. However, what if defenses block us from channeling our anger into adaptive action? If the patient’s anger triggers too much anxiety, he may use a defense to manage anxiety. Angry with the boss, but unable to own it, suppose he projects his anger onto the boss (defense mechanism). This does not reduce his anxiety. In fact, now he is afraid of his boss’ imaginary anger. As a result, he can’t channel his anger into effective assertion. This fear, dysregulated due to a projection, cannot come down until the projection comes down (see Table 2, “fear due to projective anger”). Further, let’s suppose he becomes further enraged at this boss who supposedly wants to hurt him, the anger toward this projection will be limitless too (see Table 2, “anger due to a projection”), dysregulated, and cannot come down until projection comes down.

**Table 2:** Defensive affects.

	Original emotions			Defensive affects		
	Emotions	Dysregulation	Therapeutic action	Defensive affect	Dysregulation	Therapeutic action
Anger	“Normal anger”	No	Express	“Anger in response to a projection”	Yes	Block
				“Fear due to projective anger—the supposedly angry boss”		

Grief/crying	“Good crying”	No	Express	“Weepiness”	Yes	Block
				“Protest crying”		
				“Infantile crying”		
				“Anger to cover grief”		

Guilt	Healthy guilt	No	Express	“Neurotic guilt”	Yes	Block


Thus, when patients use defenses that create defensive affects, blocking the defense is the first step to regulate the DAS. Restructuring the defenses (Frederickson, 2013) can be helpful for such patients. In our example, if the patient can learn to see the boss, rather than his projection, the projection drops, and the fear or anger resulting from the projection drops as well.

In sum, original (primary) emotions, elicited by and proportional to the stimulus, lead to a proportional, adaptive emotional response. Emotions due to real stimuli are usually limited in activation and time (Grecucci and Job, 2015). For instance, a boss makes an unfair comment that lasts 10 s. In response, the patient feels angry for 10 s. This allows him to respond adaptively to the problem. Then the anger drops, having fulfilled its function. However, when patients use defenses (for example, projection), the resulting defensive affects will be proportional to the defense (projection), not to the stimulus itself. Thus, a projection, which lasts 5 h, causes anger for 5 h. This is why defensive affects have a different shape of activation. Emotions triggered by a stimulus in reality have the shape of a wave: they rise after the stimulus and fall after the stimulus has ended or adaptive action has occurred. Defensive affects rise rapidly in response to a defense like projection and remain high as long as the patient uses the defense (in this example, projection). In Table 2, we report different examples, and we outline optimal therapist responses to regulate patients’ emotions.

## Experiential-Dynamic Emotion Regulation

In this section, we describe Experiential-Dynamic Emotion Regulation (EDER) as a set of core concepts and associated therapeutic prescriptions for the treatment of DASs. EDER is connected with a psychodynamic theory of mind and in particular with some observations derived from Intensive Short-Term Dynamic Psychotherapy (ISTDP), proposed in the 1970’s by Davanloo (1990, 2000) and further developed by others (Coughlin della Selva, 1996; Ten Have-de Labije and Neborsky, 2012; Frederickson, 2013; Abbass, 2015). Based on psychodynamic theories described in the previous section, ISTDP starts with the assumption that stimuli in life trigger emotions, which trigger anxiety and defenses. The unconscious defenses cause the symptoms and presenting problems from which patients suffer. Since anxiety and defenses cause the presenting problems and symptoms, the therapist helps the patient face his feelings – what makes him anxious – and let go of the defenses, which cause his symptoms. So, like other psychodynamic therapies, ISTDP relies on defense analysis and transference analysis to access feelings, which are avoided through defenses and the transference. (Note that interventions which mobilize feelings quickly are especially useful for high functioning patients with mature defenses. In contrast, lower functioning patients with primitive defenses require a slower exposure to feelings accompanied by anxiety regulation, plus deactivation of defenses that compromise reality testing. The patient’s levels of affect tolerance, anxiety regulation, and reality testing determine the therapeutic strategy that would be most helpful.)

### Core Principles of Experiential-Dynamic Emotion Regulation

EDER methodology can be expressed in three core principles (Grecucci, 2012; Grecucci et al., 2015, 2016a, 2017; Dadomo et al., 2016).

#### Regulation Versus Expression

To reduce a pathological affective state, we need to either: (a) promote the full expression of a true feeling, or, (b) down regulate anxiety and deactivate the defenses that create a DAS, and then promote the expression of the warded off true feeling. Once anxiety is regulated or a defensive affect is deactivated, patients must fully experience and express their adaptive feelings (Grecucci, 2012; Grecucci et al., 2015). We clearly distinguish what must be expressed (adaptive feelings) from what must be down-regulated (anxiety) or deactivated (defensive affects). When a feeling is accompanied by excessive anxiety, the therapist must regulate the anxiety to end the state of dysregulation. Then the therapist promotes the full experience and expression of the adaptive feelings. When a defense creates a defensive affect, the therapist must deactivate the defense, and then the defensive affect will disappear.

#### Focus on Emotional Experience

Constantly focus on and process emotions and dysregulatory mechanisms during the session. While patients experience an emotion, the therapist helps them observe dysregulatory mechanisms, regulate anxiety, and deactivate the defenses that create DAS moment by moment. The therapist encourages the experience of adaptive feelings but not the experience of defensive affects.

#### Use of Experiential Strategies

Experiential strategies act not only at the cognitive level, but facilitate the full experience of emotions while reducing or blocking dysregulatory mechanisms (anxiety and defensive affects). The patient is encouraged to experience emotion (Davanloo, 1990; Coughlin della Selva, 1996; Davanloo, 2000; Frederickson, 2013), rather than avoid them through cognitive or attentional strategies. During the phase of promoting feeling experience, cognitive strategies ward off feelings and are counterproductive. For example, it has been shown that in certain circumstances “reappraisal” can increase rumination (Ray et al., 2005), while rationalizing about the emotion (Freud, 1936), or “distraction” can avoid the experience of emotion (Freud, 1959a,b). Thus, when the therapist blocks defenses which prevent the patient from becoming aware of and experiencing his feelings, cognitive-attentive strategies may be detrimental. Since cognitions ward off feelings, we focus on the feelings underneath, not the cognitions. The goal of experiential techniques is not to restructure cognitions, but to help patients let go of cognitions as a defense so they can face the feeling underneath. Then patients can feel and deal rather than detach and defend. Once patients experience their original emotions, it is possible to show the causality of feelings triggering anxiety, then defenses, and then symptoms. Then we show how that pattern of causality plays out in the past, current, and therapy relationships (Malan, 1979). This process integrates their cognitions with affective experience.

### Methodology of Experiential-Dynamic Emotion Regulation

Building on previously described core principles, a general methodology for a dynamic emotion regulation can be designed as follows (see Tables 3, 4)^1^ (Grecucci, 2012).

**Table 3:** Phases and steps of the EDER methodology.

Phases	Steps for each Phase
(1) Emotion elicitation	(a) Ask for a specific example
	(b) Invite feelings.
	(c) If necessary, regulate anxiety and then invite feelings again.
	(d) Identify and help the patient let go of defenses that block the emergence of feeling.

(2) Regulatory mechanism enhancement (Awareness, attention and causality)	(a) Enhance awareness of the stimulus (b) Enhance observing capacity.
	(c) Pay attention to feeling.
	(d) Differentiate feeling from anxiety and defenses.
	(e) Understand causality (feelings→anxiety→defenses→symptoms).

(3) Dysregulatory mechanisms reduction or blocking (DAS)	(a) Understand causality of anxiety (feelings emerge→anxiety rises→DAS)
	(b) Reduce anxiety (restructure the pathway of anxiety discharge)
	(c) Understand causality of dysregulated affects (feelings→anxiety rises→defenses→defensive affects→DAS)
	(d) Block and restructure defenses which cause defensive affects

(4) Full emotional experience and elaboration	(a) Label the true feeling (subjective level).
	(b) Experience the feeling physically in the body.
	(c) Experience the impulse physically in the body.
	(d) Express the feeling (portray the associated impulse-action).


**Table 4:** Experiential-dynamic techniques to regulate emotions.

Process	Target and scope	Strategies	Model of therapy and references
Anxiety regulation	Enhancing awareness of the physiological signs of anxiety in the body	Identification, Enhancing bodily awareness, differentiating feeling from anxiety, introducing isolation of affect, changing the pathway of unconscious anxiety discharge	ISTDP (Davanloo, 1990, 2000; Coughlin della Selva, 1996; Frederickson, 2013)

Defense restructuring (experiential)	Undo the defense that creates dysregulated affects	Blocking the defense, identifying the defense, clarifying the price of the defense, clarifying the function of the defense, pointing out causality, differentiating reality from fantasy, then focusing on the true feeling that is underneath the defense	ISTDP (Davanloo, 1990, 2000; Coughlin della Selva, 1996; Frederickson, 2013)

Defense Restructuring (cognitive)	Promote meta-cognition	Point out cognitive errors	Mentalization (Bateman and Fonagy, 2006); ISTDP (Davanloo, 1990, 2000; Coughlin della Selva, 1996; Frederickson, 2013)

Emotion recognition	Enhancing awareness of emotions	- Identification, Labeling - Enhancing bodily awareness - Helping to observe emotions - Differentiating feelings from anxiety and defenses - Differentiating true feelings from defensive affects	Emotion Focused Therapy, EFT (Greenberg and Watson, 2005) AEDP (Fosha, 2000) ISTDP (Davanloo, 1990, 2000; Coughlin della Selva, 1996; Frederickson, 2013)

Emotion expression	Enhance capacity to express feelings while feeling them	- Experiencing feeling physically in the body - Experiencing the impulse physically in the body	ISTDP (Davanloo, 1990, 2000; Coughlin della Selva, 1996; Frederickson, 2013)
		- Building affect tolerance	
		- Imaginative portraiting of the impulse	


#### Phase 1: Emotion Elicitation

To elicit the precise emotion that causes affective dysregulation, the therapist asks for a specific example. In response, the patient often offers defenses (e.g., rationalizing thoughts) rather than a specific example where his feelings were dysregulated. The therapist helps patients see and let go of these defenses until they offer a clear, specific example (Frederickson, 2013). This task requires specific skills: maintaining an effective focus, recognizing defenses, regulating anxiety when necessary, identifying the price of the defenses, and encouraging the patient to stay on task: offering a specific example (see Table 3 and Frederickson, 2013, for a review of these techniques).

#### Phase 2: Regulatory Mechanism Enhancement

Once the patient offers a specific example, the therapist explores feelings. In response, the patient responds with either anxiety or defenses. By assessing the activation of the somatic and autonomic nervous systems described above in Table 1, the therapist can: (1) assess when anxiety is too high: it goes out of the somatic nervous system into the parasympathetic branch of the autonomic nervous system; (2) know when affect is dysregulated by anxiety: feeling is accompanied by the symptoms generated by the parasympathetic nervous system; (3) differentiate physiological activation of feeling from anxiety symptoms to help the patient become more regulated; and (4) assess the degree of anxiety the patient is suffering by noticing the physical symptoms: low-somatic nervous system activation; too high–parasympathetic nervous system activation.

#### Phase 3: Dysregulatory Mechanism Reduction or Blocking

Once the emotion has been unconsciously elicited in session, anxiety and defenses will result. Patients with DASs will experience either excessive anxiety which dysregulates or defenses which create dysregulated defensive affects. In this phase, the therapist may: (1) helps patient regulate their anxiety so true feelings can be experienced; (2) block the defenses that ward off feelings; (3) help patients differentiate feeling from anxiety and from defense (4) help patients differentiate a true feeling from a defensive affect. Dynamic-experiential techniques can be used to regulate anxiety or deactivate dysregulating defenses that cause DAS (See Table 4; Frederickson, 2013).

#### Phase 4: Full Emotional Expression and Elaboration

Once the DAS are resolved, the underlying true feelings which trigger anxiety should be fully experienced and expressed. During phase four, patients must be encouraged to experience their emotions in the body and to express the associated impulse (see Davanloo, 1990; Coughlin della Selva, 1996; McCullough, 1997; Davanloo, 2000; McCullough et al., 2003; Frederickson, 2013). Then patients can feel their feelings without being dysregulated.

## Conclusion

In the present paper, we explored the issue of emotion regulation inside psychodynamic approaches and how concepts of anxiety and defenses may be useful to understand patients’ DAS and we provided a framework and methodology for an Experiential-Dynamic Emotion Regulation Methodology (EDER) that incorporate emotion regulation science principles into psychodynamic psychotherapy.

A number of studies in the field of experimental psychology and affective neuroscience have collected evidence that emotion regulation strongly contributes to psychological health (Aldao et al., 2010; Hu et al., 2014) and that some strategies produce better health outcome than others (Gross, 2002; Gross and John, 2003). Here, we extend these studies by considering another important element that determines the adaptiveness of emotion regulation: the differentiation between original emotion and DAS. Physiological emotions should be expressed and a regulatory function is not necessarily required, whereas dysregulated states require regulation due to their negative contribution to individual adaptation to the environment. At the clinical level, this has important implications. The therapist should promote the expression of original emotions and block DASs.

Thus, the concepts of emotion regulation and dysregulation are consistent with the psychodynamic view of affective disorders and their therapy. In addition to cognitive-behavioral methods of emotion regulation, we include psychodynamic forms of emotion regulation. We noted that the use of techniques that implicitly involve emotion regulation could be more effective than the use of voluntary emotion regulation strategies. Indeed, in some cases voluntary emotion regulation strategies may compromise the free expression of original emotions.

Despite the explicit reference to ISTDP, we consider that the EDER concepts and techniques may be usefully incorporated into any other psychotherapy models when working with patients suffering from DASs. EDER principles offer a trans-theoretical approach for the understanding of situations that require the building of the capacities for emotion tolerance and regulation, and anxiety regulation. EDER techniques may be used to enhance emotion tolerance as an alternative to cognitive control techniques that may be detrimental when they support emotion avoidance.

Despite such interesting insights concerning theoretical models of psychotherapy, the clinical recommendation of EDER would require an empirical evaluation of the efficacy and tolerability of the model in clinical studies. Other brief psychodynamic approaches have been consistently affirmed as evidence-based therapies (Abbass et al., 2006, 2009; Driessen et al., 2010). Comparative studies are strongly recommended to add to the scientific evidence for the EDER approach, and to identify its specific change mechanisms when compared with other psychodynamic approaches.

## Author Contributions

All authors listed have made a substantial, direct and intellectual contribution to the work, and approved it for publication.

## Conflict of Interest Statement

The authors declare that the research was conducted in the absence of any commercial or financial relationships that could be construed as a potential conflict of interest.

## Footnotes

1 This methodology for Experiential-Dynamic emotion regulation can be integrated in approaches other than psychodynamic.
